# Laparoscopic Beger procedure for the treatment of chronic pancreatitis: a single-centre first experience

**DOI:** 10.1186/s12893-020-00750-7

**Published:** 2020-04-29

**Authors:** He Cai, Yunqiang Cai, Xin Wang, Bing Peng

**Affiliations:** 1 Department of Pancreatic Surgery, West China Hospital, Sichuan University, No. 37, Guo Xue Xiang, Chengdu, 610041 Sichuan China; 2 Department of Minimal Invasive Surgery, Shangjin Nanfu Hosptial, Chengdu, China

**Keywords:** Laparoscopic, Beger procedure, Chronic pancreatitis

## Abstract

**Background:**

The Beger procedure is a common surgical option in the management of the unremitting abdominal pain of chronic pancreatitis (CP). As an organ-sparing surgery, it might be a better choice than pancreatoduodenectomy (PD). However, it is rather challenging for surgeons to perform the Beger procedure laparoscopically, especially for patients with CP; indeed, it has rarely reported. Here, we describe the technique and results of our early experience in laparoscopic Beger procedure for the treatment of CP.

**Case presentation:**

Five patients (1 male) with CP (alcohol induced, *n* = 3; idiopathic, *n* = 2) who underwent laparoscopic Beger procedure from May to October 2019 in West China Hospital were included in this study. The median pancreatic duct diameter was 6.8 (4 to 12) mm. The median operating time was 275 (150 to 305) minutes without conversion. Only one patient (20%) developed a grade B pancreatic fistula. One patient required re-operation for jejunal anastomotic bleeding on the first post-operative day. The median hospital stay was 11 (9 to 34) days. No patient experienced biliary fistula, gastroparesis, duodenal necrosis, or abdominal bleeding. The 90-day mortality rate was 0%. All the patients were pain free in the two months after the operation.

**Conclusion:**

The laparoscopic Beger procedure is feasible and safe with good short-term results and some potential benefits in selected patients with chronic pancreatitis. Further study and longer follow-up are required.

## Background

Chronic pancreatitis(CP) is a recurrent fibro-inflammatory disease of the exocrine pancreas leading to permanent structural impairment of the parenchyma [1]. Patients with CP mostly present with inflammatory pancreatic head enlargement, with possible development of local complications such as intractable pain, stenosis of the pancreatic or bile duct, large and numerous pancreatic stones, or compression of retro-pancreatic vessels [2]. In those patients, the need for surgical intervention is greater than the need for endoscopic treatment, which has been proven in randomized controlled trials to especially address pain relief [3–5].

Surgery for chronic pancreatitis can be broadly categorized into drainage or resection procedures [6]. The Beger procedure is a combined drainage and resection technique that was first introduced by Beger and colleagues for CP in the early 1970s [7]. The idea was that a subtotal resection of the pancreatic head might eliminate complications related to the inflammatory head mass, while preserving the duodenum, bile duct, normal enteric passage, interaction of duodenal hormones and insulin secretion [8]. With the development of minimally invasive surgery, this approach can provide several advantages over open surgery, such as fewer complications and faster recovery. However, performing the Beger procedure laparoscopically is technically challenging, especially for CP. There are only a few case reports of the minimally invasive Beger procedure for CP in the literature [9, 10]. Herein, we report our early experience in laparoscopic Beger procedure for the treatment of CP.

## Case presentation

### Patients

This study was conducted from May to October 2019 in the Department of Pancreatic Surgery of West China Hospital. Five patients (1 male, 4 females, median age 52 years, range 29–73 years) with an established diagnosis of CP who underwent laparoscopic Beger procedure were included in the study (Table 1). The preoperative symptoms included repeated abdominal pain (5 cases), pancreatic duct stones (4 cases), diarrhoea (1 case) and weight loss (2 cases). The diagnosis was confirmed by enhanced computed tomography scan. The main indication for surgery was pancreatic head enlargement with intractable pain (5 cases), and both medical management and endoscopic interventions fail to provide pain relief. Other indications for operation include obstruction of the common bile duct, pancreatic duct obstruction or stenosis, duodenal obstruction, and/or entrapment of the retroperitoneal superior mesenteric or portal veins. All patients were informed of the surgery, and signed consent forms were obtained from them. The median (range) pancreatic duct diameter were 6.8 (4 to 12) mm. The preoperative details and disease characteristics are shown in Table 1. Pain was assessed by the validated Izbicki [11] pain score, as depicted in Table 2.

**Table 1:** Preoperative Details and Disease Characteristics

Patient	Etiology	Age/Sex	MPDD (mm)	Comorbidities
1	Idiopathic CP	29/F	9	Pancreatic endocrine insufficiency
2	Alcoholic CP	73/M	12	Pancreatic endocrine and exocrine insufficiency
3	Alcoholic CP	35/F	8	Nil
4	Alcoholic CP	56/F	6	Nil
5	Idiopathic CP	52/F	4	Nil

CP indicates chronic pancreatitis; *F* female, *M* male, *MPDD* main pancreatic duct diameter

**Table 2:** Preoperative Pain Score (Total Score: Sum of Single Values Divided by 4)

Patient	Frequency of pain attacks^a^	VAS^b^	Analgetic medication^c^	Inability to work^d^	Total
1	75	50	20	50	48.75
2	50	40	15	25	32.5
3	100	50	20	75	61.25
4	50	60	15	50	43.75
5	75	40	20	50	46.25

^a^Frequency of pain attacks: Daily (100)/ Several times a week (75)/ Several time a month (50)/ Several time a year (25)/ None (0)

^b^VAS (Visual Analog Scale of Pain): (No pain: 0—imaginative maximum of pain: 100)

^c^Analgetic medication (morphine-related analgetic potency): Morphine (100)/ Buprenorphine (80)/ Pethidine (20)/ Tramadol (15)/ Metamizole (3)/ Acetylsalicylic acid (1)

^d^Inability to work (last year): Permanent (100)/ ≤1 year (75)/ ≤1 month (50)/ ≤1 week (25)/ None (0)

### Surgical procedures

**Fig. 1:**
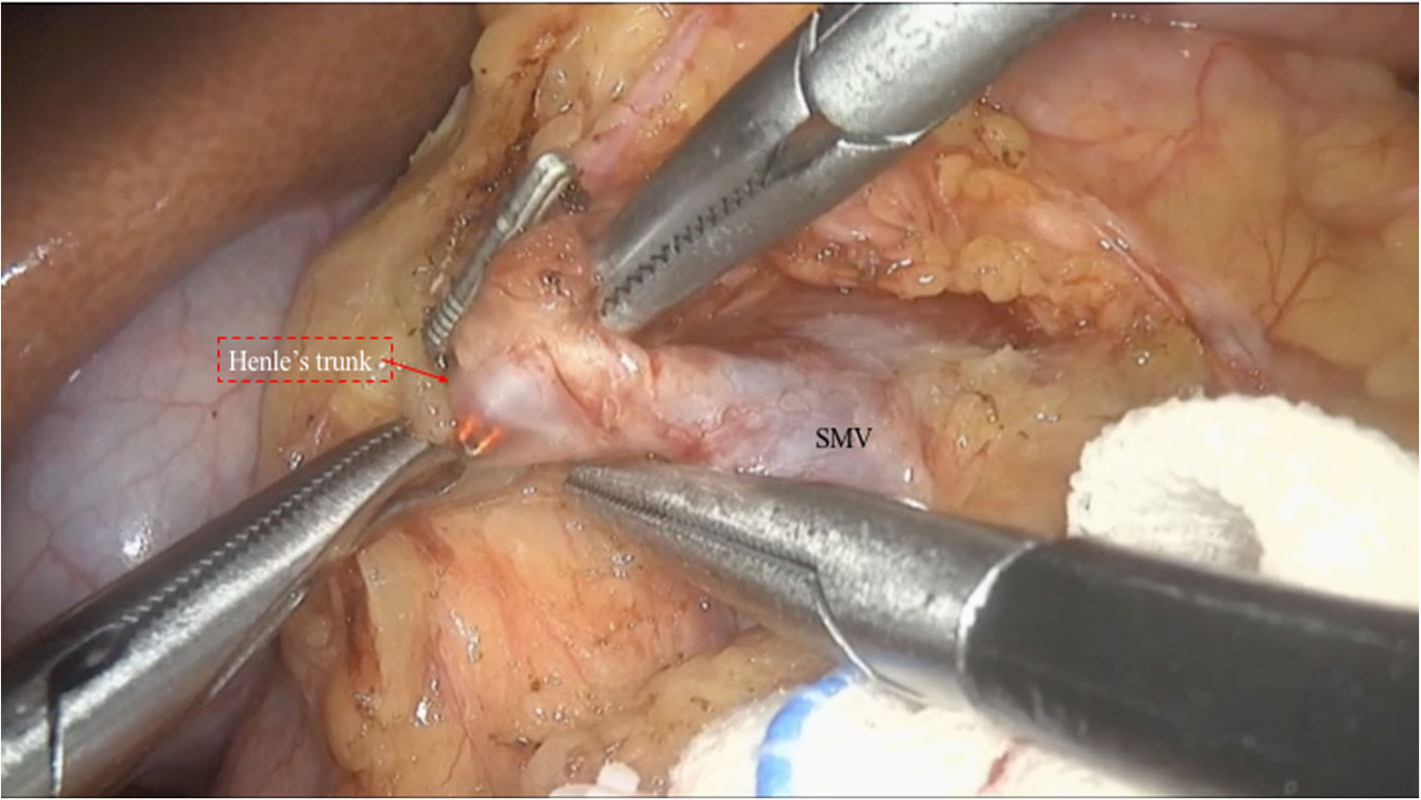
Dissect the Henle’s trunk

**Fig. 2:**
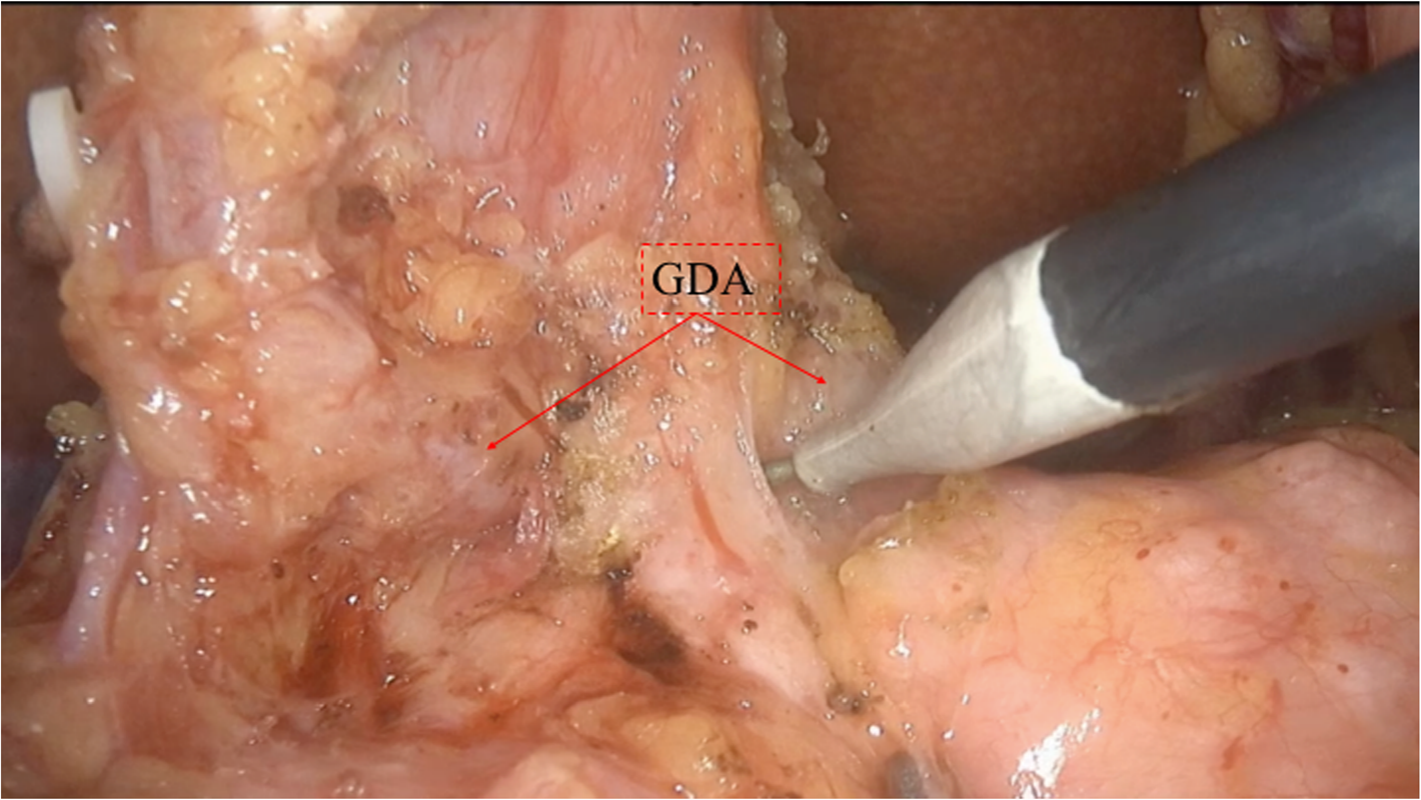
Identify and protect the GDA

**Fig. 3:**
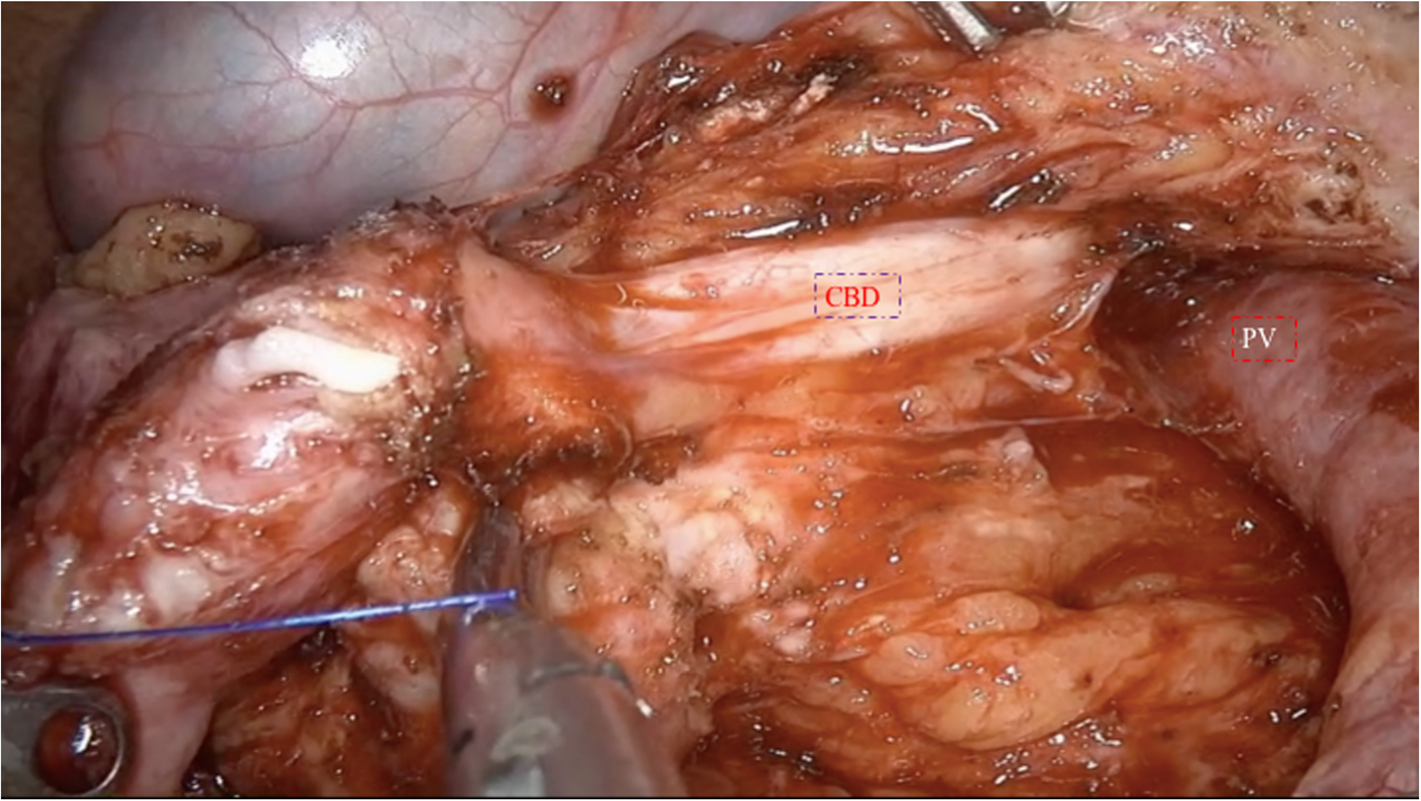
Identify and protect the CBD

**Fig. 4:**
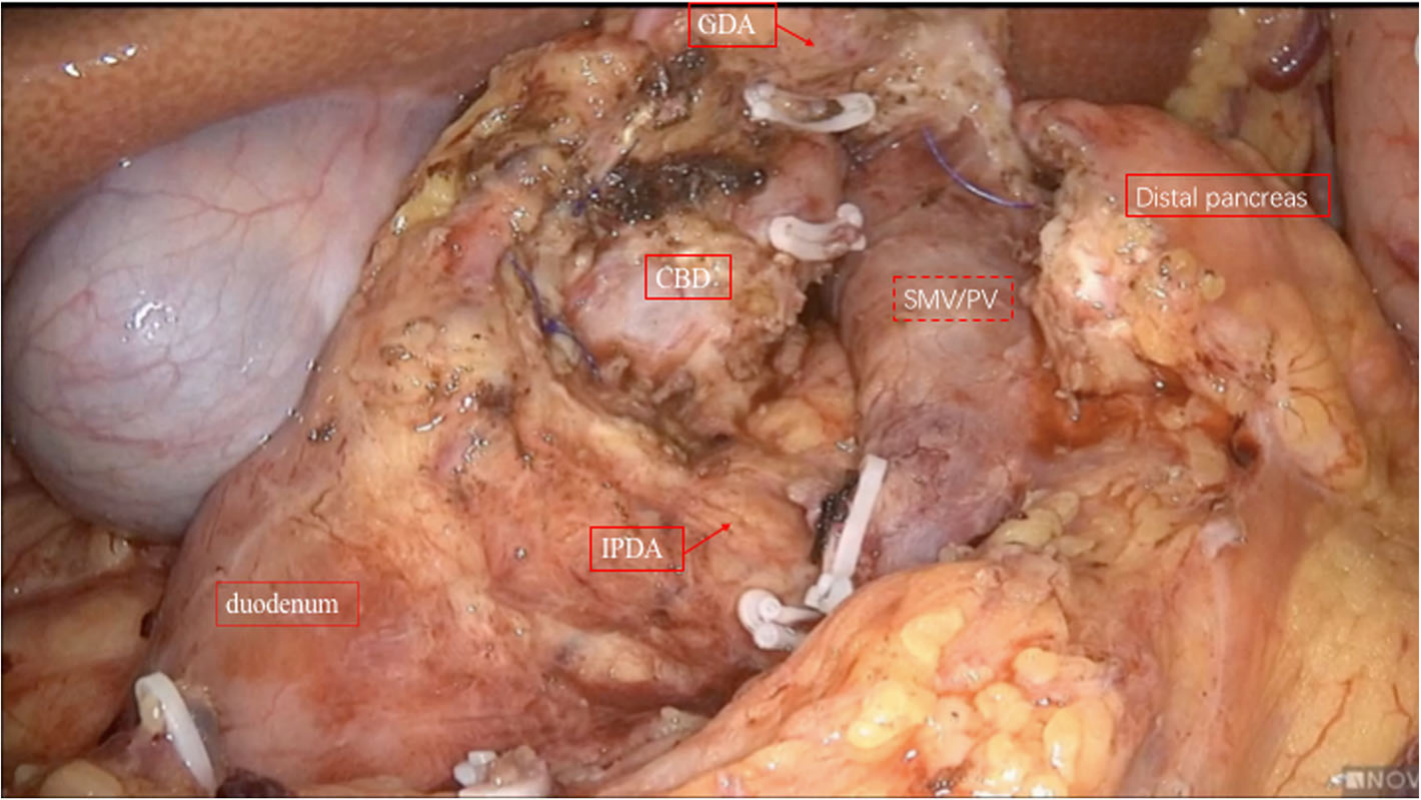
The complete laparoscopic Beger procedure of while preserving the duodenum, biliary tract and pancreatic duodenal arterial arcades

**Fig. 5:**
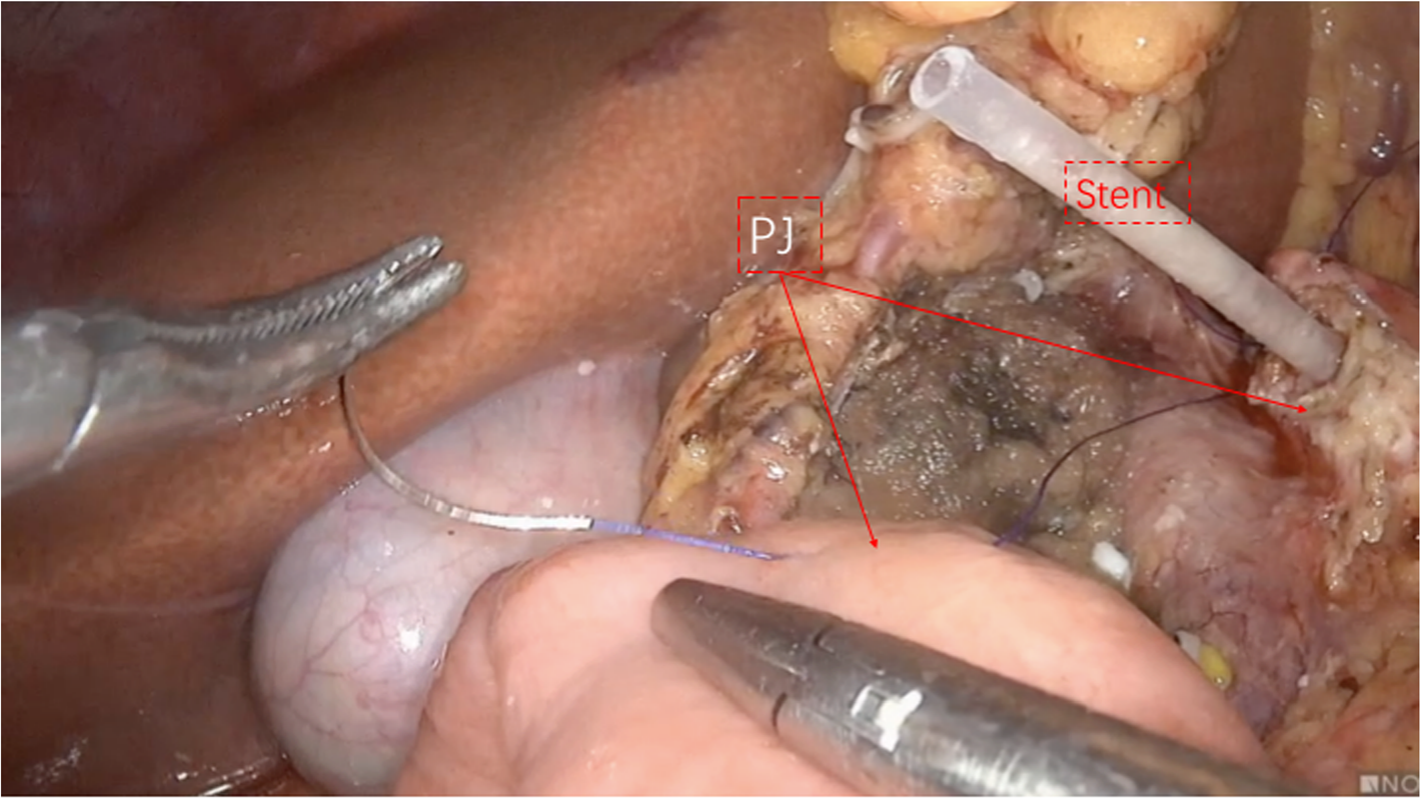
Bing’s Duct-to-mucosa pancreaticojejunostomy

The patients were placed in the supine position with the two legs apart. The observing trocar (10 mm) was located at the inferior umbilicus. Four trocars were distributed symmetrically at the midclavicular line and anterior axillary line. Another 5-mm trocar located at the subxiphoid was used to suspend the stomach. The gastrocolic ligament was opened and the hepatic flexure of the colon was taken down to explore the head of the pancreas. The right gastroepiploic vein and Henle’s trunk were dissected (Fig. 1). The no. 8a lymph node was dissected for intraoperative rapid frozen pathology. The pancreatic neck was transected with an ultrasonic scalpel, and the main pancreatic duct was transected with cold scissors. The superior mesenteric vein (SMV) was retracted to the left. The uncinate process of the pancreas was retracted to the right, and subcapsular dissection was carried out, paying particular attention to protecting the inferior pancreaticoduodenal artery (IPDA) and its branches. Then the gastro-duodenal artery (GDA) was identified and protected (Fig. 2). The anterior superior pancreatic duodenal artery (ASPDA) was dissected. The upper part of the pancreatic head was separated to expose the common bile duct (CBD) (Fig. 3). The pancreas was dissected from the left edge of the duodenum and the right edge and the ventral edge of the CBD (Fig. 4). The posterior superior pancreatic duodenal artery (PSPDA) was identified at the dorsal edge of the CBD. The PSPDA and its branches were carefully preserved. The proximal of main and accessory pancreatic ducts were identified and sutured. Roux-en-Y duct-to-mucosa pancreaticojejunostomy was carried out with the left pancreas which was performed using the technique of Bing’s anastomosis (Fig. 5) [12]. Two closed drainages were routinely placed behind the pancreaticojejunostomy and near the common bile duct, respectively.

### Operative and postoperative outcomes

The operations were successfully performed for the five patients without conversion. The operative and postoperative outcomes are shown in Table 3. The operative times varied between 150 and 305 min (median 275 min). Blood loss ranged between 100 and 300 ml (median 200 ml). Hospital stay varied between 9 and 34 d (median 11 d). Only one patient (20%) developed a grade B pancreatic fistula [13] with drainage delayed on the thirtieth day after the operation. One patient required re-operation for jejunal anastomotic bleeding on the first post-operative day, she was discharged uneventfully on the tenth day after the second operation. No patient experienced grade C pancreatic fistula, biliary fistula, gastroparesis, duodenal necrosis, or abdominal bleeding. No mortality had occurred at 90 days after the surgery. All patients were pain free in the two months after the operation. No statistical comparison was performed due to the small sample size.

**Table 3:** Operative and postoperative outcomes

Patient	Operative Time (min)	Blood loss(ml)	Hospital Stay (d)	Reoperation	Complications
1	150	100	14	0	0
2	275	300	11	0	0
3	230	200	34	0	Grade B pancreatic fistula
4	300	200	10	Laparoscopic re-anastomosis for jejunal anastomotic bleeding	0
5	305	200	9	0	0
Total	275(190–302.5)^a^	200(150–250)^a^	11(9.5–24)^a^	1(20%)	1(20%)

^a^data shown as median (quartile)

## Discussion and conclusions

CP is a complex inflammatory disease involving an enlarged pancreatic head as the main morphologic pathology and pain as the most common symptom [6]. Pain reduction is the main goal of treatment because it is the most important factor that influences a patients’ quality of life [14, 15]. The pain is caused by the obstruction of the pancreatic duct and neuronal alterations in the fibrous converted pancreatic tissue in which the pancreatic head is the pacemaker [16]. Therefore, surgery for CP can be broadly classified into three categories: drainage procedures, partial pancreatic resection and their combination. The most common drainage procedure is the modified Puestow procedure, also known as lateral pancreatojejunostomy [17]. The first duodenum preserving pancreatic head resection (DPPHR) was introduced by Beger in 1972 and consisted of preserving a thin rim of pancreatic tissue along the duodenal loop and two pancreatic anastomoses [7]. Other DPPHR procedures, including the Frey [18] and Berne [19] procedures related to the removal extent of pancreatic tissue or the combination with drainage aspects, have been suggested. For many years, pancreaticoduodenectomy (PD) has been applied as a standard operation in the treatment of CP [15]. Compared to DPPHR, PD is a radical surgery associated with multi-organ resection and may result in endocrine and exocrine dysfunctions of the pancreas. DPPHR is more technically challenging and time-consuming. Beger procedures should be considered in patients with an enlarged pancreatic head (> 4 cm) [15]. For patients with painful CP, a dilated main pancreatic duct (> 5 mm) and a normal-sized pancreatic head (< 4 cm), a lateral pancreatojejunostomy and Frey’s procedure may be suggested.

In contrast to the classic Beger procedure, we only preserved a small part of pancreatic tissue behind the CBD; therefore, only one pancreatic anastomosis was necessary [7]. Moreover, compared with open surgery, laparoscopic surgery can more clearly show the vascular arcades due to the amplification of the surgical field. We preserved the IPDA and PSPDA; however, we resected the ASPDA, which is also different from the classic Beger procedure [7]. Nonetheless, laparoscopic surgery for CP is rather challenging with regard to fibrous scarring, and we completed all surgeries successfully without conversion. In an early study, Yin Z reported better postoperative pain relief and improved quality of life with the Beger procedure compared with PD [20]. After a 15-year long-term follow-up, Kai Bachmann found equal pain control but better quality of life for the Frey procedure had versus PD in the treatment of CP [21]. Diener [22] carried out a multicentre randomized controlled trial that included 250 CP patients with 24 months of follow-up after surgery, and the result showed that the quality of life was equal between partial PD and DPPHR. Although some doubt remains, DPPHR (including the Beger procedure) seems to be the operation of choice for patients with CP [15].

No differences are seen for DPPHR versus PD regarding postoperative pain relief, overall mortality and morbidity up to two years of follow-up [22]. A Dutch study including 146 patients with CP who underwent surgery reported pain relief in 68% of patients [23], and a 2015 meta-analysis of 23 studies reported that pain relief was achieved in 89% of patients who underwent the Frey procedure [24]. Our study showed a 100% pain-free rate. The incidence of pancreatic fistula may be higher in DPPHR than that in PD due to a pancreaticojejunostomy and a large surface of pancreatic incision on the duodenal side. In a system review, Beger et al. reported that the incidence of grade B/C pancreatic fistula was 13.6% after DPPHR [25]. The incidence of grade B pancreatic fistula was 20% in our study, and the small sample size may have led to a higher statistical rate. Bile leakage is another common complication of DPPHR. Cao J et al. reported that the incidence of biliary fistula was 16.7% after laparoscopic DPPHR [26]. No patient in our study developed grade C pancreatic fistula, biliary fistula, gastroparesis, duodenal necrosis, or abdominal bleeding.

Overall, it is rather technically challenging to perform the Beger procedure laparoscopically, especially for CP. There are only a few case reports of the minimallyl invasive Beger procedure for CP in the literature [9, 10]. Here, we elaborated the surgical techniques and outcomes of the operation in detail. Although these results need validation with studies enrolling a greater number of patients and a comparison with open surgery, our study shows that the laparoscopic Beger procedure is feasible and safe with good short-term results and some potential benefits in selected patients with chronic pancreatitis.

## Data Availability

The data supporting the findings of this study are available within the article.

## Abbreviations

CP: Chronic pancreatitis
DPPHR: Duodenum preserving pancreatic head resection
PD: Pancreaticoduodenectomy
CBD: Common bile duct
GDA: Gastro-duodenal artery
ASPDA: Anterior superior pancreatic duodenal artery
PSPDA: Posterior superior pancreatic duodenal artery
IPDA: Inferior pancreaticoduodenal artery
SMV: Superior mesenteric vein
PV: Portal vein
PJ: Pancreaticojejunostomy
